# Sports mega-events and changing world order

**DOI:** 10.1177/00207020231163063

**Published:** 2023-03-08

**Authors:** David R. Black

**Affiliations:** Department of Political Science, 3688 Dalhousie University, Halifax, NS, Canada

**Keywords:** Sports mega-events, olympics, world orders, hegemony, human rights, sanctions, conspicuous consumption

## Abstract

Processes of making, sustaining, reforming, and un-making world orders are constants in global politics and development. Understood in the neo-Gramscian tradition pioneered by Robert Cox, ideas, institutions, and material capabilities combine to shape the range of possibilities for more and less stable orders. Sports mega-events (SMEs)—most prominently, the Olympic Games—have played an underappreciated role in this process. This paper examines the ways in which the Olympics manifested and supported the rise of globalized neoliberal hegemony in the early 1980s, the reconfiguration and erosion of this order through the 1990s and 2000s, and efforts to fundamentally revise this order in the new millennium. Particular emphasis is placed on the dual role of SMEs and the Olympics as manifestations of conspicuous consumption and the pursuit of prestige on the one hand, and as focal points for sanctions campaigns and boycotts on the other.

We live in a time when the fluidity and uncertainty of future world order(s) is a pressing preoccupation. The relative decline of “the West” and what remains of the *Pax Americana*; the rise of China and, more broadly, a growing range of “illiberal” actors and forces; the tectonic shifts in the world economy wrought by the imperatives of ecological sustainability and the sudden but sustained shock of the global COVID-19 pandemic; deeply destabilizing repercussions of technology-enabled and privatized surveillance and intelligence capabilities;^1^ and now the disorienting uncertainties arising from Russia’s war on Ukraine—these and other profound changes suggest we are moving towards a broad and deep realignment of the foundations of world (dis)order.

The dynamic processes of making, sustaining, reforming, and un-making world orders are, however, constants in global politics and development. While realist accounts anchor these processes in the shifting configuration of state-based great power(s) along with their allies and acolytes, the historicist and neo-Gramscian tradition popularized by Robert Cox highlights the multi-dimensional nature of this process, in which configurations of ideas, institutions, and material capabilities combine to shape the range of possibilities for more and less hegemonic (i.e., relatively stable and consensual) orders.^2^ This process is not easy or rapid. Ordering structures change only slowly and develop an array of vested interests and theoretical approaches that seek to sustain them—what Cox famously characterized as “problem solving” as opposed to “critical” theoretical approaches.^3^ Identifying *when and how* such orders reach decisive turning points, and what they *turn towards* is therefore an essential, though challenging, analytical task, with important implications for praxis.^4^

In this paper, I argue that an underappreciated role in this unfolding process has been played by global elite sport, and in particular sports mega-events (SMEs). At the apex of this complex of cultural, ideational, institutional, and material dynamics is the modern Olympic Games, described by Susan Brownell as the “premier global ritual for expressing global community.”^5^ The Games are presided over by the powerfully enigmatic International Olympic Committee (IOC).^6^ Despite the plethora of authors and analyses of SMEs in general and the Olympics in particular, relatively little attention has been paid to the role they have played in world-order making and unmaking, as key sites for manifesting, sustaining, undermining, and contesting dominant orders. This role should not be understood as *causal*, at least in its own right, but as an important part of the *contextual fabric* within which relative stability and change unfolds. In this paper, I will focus primarily on the ways in which the Olympics^7^ supported and enabled the rise of the new phase of globalized neoliberal hegemony beginning in the early 1980s, their role in the reconfiguration and erosion of this order through the 1990s and 2000s, and their place in current efforts to fundamentally revise this order, anchored by an ascendant China and, secondarily, a revanchist Russia. In doing so, I will place particular emphasis on the role of SMEs/the Olympics as manifestations of “conspicuous consumption”^8^ and the pursuit of prestige on the one hand, and as focal points for sanctions campaigns and boycotts on the other.^9^

## SMEs, political economy, and world order

The political economy of SMEs in general and the Olympic Games in particular has generated a vast and growing scholarly and popular literature.^10^ Thematically and theoretically, this work has straddled a wide range of subjects, reflecting the multi-scalar and multi-dimensional character of SMEs. These subjects have included, among others, urban and regional development, “growth coalitions,” and global cities; neoliberal globalization and alter-globalization; securitization; branding and marketing power; human rights advocacy and popular resistance; the “soft power” strategies of governments; the ambitions of “rising states” in the global East and South; and the distinctive role and influence of international sports organizations (ISOs), exemplified by the IOC and its associated network of global sports governance institutions. As Bruce Kidd has noted, each SME spawns “multiple narratives”^11^ and, given the intensity of the process of bidding, preparing, and staging them, generates its own event-specific literature (for example, concerning Sydney 2000, Beijing 2008, Vancouver 2010, London 2012, Rio 2016, etc.). Yet, while various scholars and analysts have periodized the history of the Olympics and “Olympism”^12^ in relation to the dominant normative, political-economic, and geopolitical currents of their era, relatively little attention has been paid to their role in illuminating, constituting, and *making* (or unmaking) the order in which they are embedded.^13^

Viewed from this perspective, the Olympic Games should be analyzed less in terms of specific events, and more in terms of pivotal clusters and/or relatively long cycles which reveal processes of normative and political-economic emergence, consolidation, decay, and revision within interrelated national and world orders. These in turn are underpinned (in Coxian terms) by particular configurations of ideas, institutions, and material capabilities. In this paper, I focus on three overlapping phases of the interplay between SMEs and order-making, linked to: 1) the emergence of the post-Cold War era of “high neoliberalism” and a renewed phase of Western hegemony (as seen in the Moscow 1980 and Los Angeles 1984 Olympic Games); 2) the consolidation, adaptation, and erosion of this order (from the 1988 to the 2016 Games); and 3) the prospective revision of this order (associated especially with the Beijing Games of 2008 and 2022 and the Sochi Games of 2014, along with other SMEs like the 2018 FIFA World Cup), including the contestation surrounding them by governments, human rights groups, and ISOs. The processes by which these changes are “engineered” are both immanent (arising from within the inherent logic of particular orders) and intentional (seeking to actively instigate processes of change).^14^ Historically, the former has tended to be associated with Western capitalist states and institutions, and the latter with authoritarian and “revisionist” states and institutions within and beyond the West.^15^ In the unfolding of these processes, I pay particular attention to the prevalence of conspicuous consumption by Olympic hosts—that is, their ability to absorb and sustain spectacular displays of organizational, architectural, and infrastructural ambition with the objective of elevating their international prestige and status; and the role of sport sanctions and boycotts, including both their advocacy and their implementation, as signals of the relative influence and prestige of senders and targets and the ordering principles they favour. We begin with the sequence of events encompassed by the 1980 Moscow Olympics and the 1984 Los Angeles Games, which together reflected a pivotal turn in the post-World War II global order.

## Twilight in the USSR, “Morning in America,” and the rise of the neoliberal global order

The political importance attached to sport by the Soviet Union in pursuit of its transformative objectives, nationally, regionally, and internationally, is well known. Internationally, from its first Olympic Games at Helsinki in 1952 the USSR’s competitive success was viewed by its Communist Party elites as critical in advancing its status- and prestige-seeking objectives. Unsurprisingly, therefore, its first opportunity to host the Olympic Games in 1980 became a Soviet state priority of the highest order—an unparalleled opportunity to burnish the prestige and status of the Soviet Union, and to which the state’s emphasis on central planning, whatever its failings, was exceptionally well-suited.^16^ Thus, in contrast to many other Olympics before and after (including the Montréal Games that immediately preceded them), preparations for the Moscow Games were both monumental and well in hand long before the event itself.^17^

The Soviet invasion of Afghanistan in December 1979, however, triggered a chain of events that ultimately resulted in the US government successfully pressuring the US Olympic Committee to boycott the Games in protest, and persuading more than sixty other countries (including Canada, Japan, China, Israel, West Germany, and most Islamic countries) to follow suit. The anticipated international media attention and hundreds of thousands of visitors were both greatly diminished, with NBC forfeiting its US broadcast rights at a cost of $22 million plus a further $40 million in lost advertising revenue.^18^ Thus, although the event itself was a competitive and organizational success, Nina Kramareva and Jonathan Grix conclude that “without US participation and with the other countries following suit, the 1980 Moscow Olympics were relegated to the second rank of sports events.”^19^

Both the process by which the boycott was mobilized and its repercussions were controversial. A politically vulnerable US President Jimmy Carter felt compelled to project a strong response to the Soviet invasion. He gravitated towards the Olympic boycott strategy because of its high visibility and relatively low cost and risk in both strategic and economic terms.^20^ The process by which support for and against the boycott was mobilized was itself messy and controversial, in part because of the resilient norm that sport should remain autonomous from politics.^21^ Sports people then and now continue to echo the conclusion reached by James Riordan, one of the leading scholars of Soviet sports, that “the Moscow boycott was an abject failure,”^22^ seeking to reinforce the self-interested claim that sports boycotts never “work” and hurt only dedicated and self-sacrificing athletes.

In the case of Moscow, however, this claim only makes sense if the impact of the boycott is measured in terms of the narrowly instrumental objective of compelling the Soviet Union to withdraw from Afghanistan. This was never seen as a realistic possibility by either the Soviet Union or its antagonists, including the US administration. Understood as a painful “punishment” to support an international claim of wrongdoing however—a much more plausible justification for many sanctions measures^23^—its impact was considerably more significant. In addition, the number of countries supporting the boycott, however their support was secured, could be seen as a clear if crude measure of the United States’ relative capacity to mobilize international support compared with its Soviet rival. Moreover, the diminished Games arguably had domestic repercussions within Communist bloc countries. Riordan asserted that while “it would be a bit extravagant to blame the Moscow Olympics for the demise of communism…for many citizens of communist states (the Games) brought tensions to a head.”^24^ In short, the failure of the Games to fulfill their prestige-seeking objectives arguably tilted the balance of global normative power further away from the Soviet Union and its revisionist objectives.

To fully appreciate this result, the Moscow Games need to be situated in relation to the next Olympiad in Los Angeles in 1984. The Los Angeles Games are widely understood as a critical turning point for the Olympics when, to secure the very survival of the modern Olympics (with Los Angeles as the sole bidder for the event), both the private Los Angeles Olympic Organizing Committee (LAOOC) and the IOC were compelled to embrace a fully commercialized model, featuring television deals of unprecedented size, a commodified Olympic torch relay, and exclusive commercial sponsorships to pay for the Games and indeed (almost uniquely) render them profitable.^25^ Despite the fact that the Los Angeles Games were also the target of a significant boycott, with the Soviet Union leading fourteen communist allies in withdrawing from participation, the repercussions were very different.^26^

Whereas the 1980 Olympics had greatly diminished international exposure and stature due to the boycott, the 1984 Games dovetailed with the re-election campaign of Republican President Ronald Reagan to amplify his folksy message of a new “morning in America.”^27^ In the absence of its principal competitors, the United States won an avalanche of medals—almost three times as many as the second-place West Germans. This, combined with the jingoistic coverage of host broadcaster ABC, fuelled an outpouring of American patriotism and a sense of national renewal after the post-Watergate and Vietnam years of the 1970s. Moreover, the marketized model of the Games’ organization appeared to validate and certainly popularized private sector leadership, offering an apparent model for profitability alongside organizational spectacle and success. With the successful implementation of this new model of organization and delivery, an anachronistic mega-event which had been in deep trouble became an attractive answer for states and corporations alike seeking visibility and competitive success in an increasingly globalized world economy. Moreover, under the adroit and opportunistic leadership of Juan Antonio Samaranch, a former senior member of Francisco Franco’s Falangist regime in Spain, the IOC itself embraced this new model of commercialized, neoliberal organization. In so doing, it dramatically renewed competition to host both the Summer and Winter Games.^28^ In short, the Olympic cluster extending from the awarding of the 1980 Games to Moscow in 1974 through to the commercial, political, and ideational success of the 1984 Los Angeles Games ten years later strongly reinforced the emerging trend towards neoliberal hegemony anchored by a resurgent America. To state the obvious, the Olympics did not instigate these changes, but they did popularize and legitimate them. This trend was at first reinforced, but then increasingly questioned and contested in the generation-long Olympic Games cycle to follow.

## The rise, elaboration, adaptation, and erosion of the neoliberal Olympics

The Los Angeles Games ushered in a new era of heightened prominence and popularity for the now-commodified “Olympic movement.” From a single bidder for the 1984 Games and only two for the 1988 Olympiad, the number of bidders rose steadily to six (1992 and 1996), eight (2000), and eleven (2004), before receding to ten (2008), nine (2012), seven (2016), six (2020), three (2024), and back to one (2028 and 2032). Moreover, the range of bidders also became much more regionally and developmentally diverse, extending well beyond the Games’ Eurocentric roots to encompass new entrants from Asia, Latin America, the Middle East, and Africa.^29^

The “arc” of this newfound rise and subsequent decline in the popularity of Olympic bidding mirrored a narrative arc in which each successive event elaborated a story of the mounting ambition and success of “leveraging legacies” from Olympic hosting.^30^ This was followed by growing disenchantment as awareness of the costs and risks of Games hosting grew and with it, increasingly robust and sophisticated anti-Olympic resistance.^31^ The next Summer Olympics after Los Angeles were the Seoul Games of 1988—a geostrategically improbable choice that never would have been made without the paucity of viable alternatives when the vote took place in 1981.^32^ The Games were awarded to South Korean General Chun Doo-hwan’s repressive, modernising military regime, which clearly intended to use the Games to legitimize itself in the eyes of the world. As with other Asian SMEs, the preparations were state-led, infrastructurally ambitious, and symbolically freighted.^33^ As the Games approached, however, mass resistance, incorporating much of Korea’s rising middle class demanding democratizing reforms, mobilized in the streets. These sustained protests in the glare of unprecedented international media attention raised for Korea the spectre of the unimaginable humiliation of losing the Games. In this context, the regime conceded virtually all of the liberalizing demands of the opposition in June 1987, paving the way for “free and direct presidential elections, the protection of civil liberties and a widespread political amnesty.”^34^ This clearly signalled that the Games could be linked to growing political freedoms as well as economic liberalization—suggesting in good neoliberal fashion that all good (i.e., liberal) things really *could* go together. In fact, subsequent Games have largely belied this promise, but the Seoul Games went a long way toward cementing the rising fortunes of the Games in the context of a rising neoliberal tide. They also associated the Games with upward development mobility and prosperity, as Korea effectively announced to the world its transition from the impoverished, war-torn “developing country” of 1953 to one that, by 1996, would become a member of the club of rich developed countries in the Organization for Economic Cooperation and Development (OECD)—a span of less than two generations.

The 1992 Games in Barcelona, staged in IOC president Samaranch’s home city, came to be widely viewed as a success for at least three main reasons. First, as the initial post-Cold War Games, they were also the first since Munich in 1972 (itself scarred by the infamous “Munich massacre” of Israeli athletes and officials) to avoid a significant boycott. Indeed, they were distinguished by the return of a South African team for the first time since 1960, composed for the first time ever of athletes of all races, even while South Africa was still navigating its difficult transition to majority rule. Thus, Barcelona achieved the IOC’s highest aspiration of “universality.” Second, they were distinguished by the presence of the United States’ basketball “dream team,” whose exceptional global star power validated the promise of a fully professionalized Olympics. Third, and most importantly, they exemplified the use of the Games for urban regeneration, place branding, and international tourism promotion—all firmly aligned with the neoliberal formula for competitive economic success in a globalizing world economy. In the words of David Goldblatt, a city that “just a decade earlier, had been a post-industrial backwater, a Mediterranean rustbelt port, was reinvented for the rest of the world as a cultural centre, an architectural jewel, a leading tourist destination and an exemplar of modern high-density urbanism.”^35^ In short, the Games were associated with the promise of a new era of urban regeneration, national prosperity, and upward mobility aligned with a neatly packaged celebration of collective identity—in this case both Catalan and Spanish.

The 1996 Atlanta Games marked a setback in the upward march of SME-driven ambition and achievement. They were widely seen as tackily over-commercialized and, because of their over-dependence on the private sector (“a modernized version of Los Angeles 1984,” according to Goldblatt), were infrastructurally disorganized and under-prepared. They were also associated with heretofore unprecedented levels of displacement of the urban poor, combined with the creation of urban infrastructure (such as the new Centennial Olympic Stadium, subsequently renovated as a professional baseball stadium for Atlanta Braves and CNN owner Ted Turner) that transparently benefited the already super-rich. None of this was in itself surprising; Atlanta’s failing was that it was too transparent and too extreme. The result was not (yet) to discredit the neoliberal model, but rather to enhance the popularity of public-private partnerships, which could mitigate the extremes of reliance on the private sector while shielding major Games-driven public expenditures from the constraints of normal levels of public transparency and accountability.

Sydney narrowly defeated Beijing for the right to host the 2000 Games, playing on human rights concerns in the shadow of the Tiananmen Square massacre while pursuing an aggressive and targeted strategy of delegate lobbying, and favours, to achieve this result. The Games were noteworthy for a heightened level of anti-Games activism and resistance in the preparation phase, reflecting the growth of a transnational anti-globalization movement that had risen to prominence in the Olympic context with Toronto’s Bread Not Circuses coalition, which was instrumental in scuttling that city’s bid for the 1996 Games. Despite the controversies in the run-up to the Games, however, the event itself was widely regarded as well-organized and successful in projecting a more diverse and “global” Australia, as well as one more committed to reconciling with Indigenous Australians. Sydney further reinforced Australia’s already-firm foundation for future SME bids and an events-centred development strategy. It also sought to project itself as a “Green Games,” reflecting the post-1992 effort to promote a “green modernisation” approach to sustainable development that portrayed economic growth and environmental sustainability as complementary.^36^ In Sydney’s case, its green claims were primarily manifested in the remediation of a huge post-industrial wasteland to create the Sydney Olympic Park. While the Park is “still searching for a soul,”^37^ it has not been saddled with the sort of derelict white elephant venues—“world class” facilities left to decay—that have afflicted many other hosts. Reflecting the growing stakes and risks of SMEs, one commentator recently concluded that, “Most importantly, Australia avoided the huge reputational hit of getting the Games ‘wrong.’ Only four years earlier, the 1996 Atlanta Olympics were widely criticised for being poorly organised and over-commercialised…”^38^

While it was not apparent at the time, Sydney represented a high-water mark for the neoliberal Games and their benign associations. Even as the post-Cold War era of “high neoliberalism” and “third way” Western hegemony^39^ was abruptly punctured by 9/11 and its securitized aftermath, the growing excesses of Games bidders and hosts, the financial and social unsustainability of the events and their “legacies,” and the obvious inequities which (despite their developmental promises) were routinely reinforced led to growing resistance and skepticism towards their “magical” claims.^40^ Against long odds, the Athens Games in 2004 miraculously succeeded in readying their exceptional venues in time to deliver what was perceived in the moment as an “epoch-defining Games.” The price, however, was dramatically inflated costs that were instrumental in plunging Greece into a deep and protracted economic crisis.^41^ In their aftermath, the Athens Olympics became a watchword for derelict white elephant venues. Immediately prior to this, the IOC itself was plunged into crisis by a bribery scandal implicating the Salt Lake City Winter Olympics organizers. While the resulting reforms succeeded in “righting the ship,” they left a lasting impression of a privileged, entitled, and unaccountable organization—an impression that stuck to both the IOC and the Games they were the custodians of.

We will circle back to the 2008 Beijing Games in the next section, highlighting an emergent trend towards authoritarian and illiberal hosts in the face of the “low-intensity democracy” promoted by Western governments and institutions in the post-Cold War era. The 2012 London Games, orchestrated in the heartland of third-way capitalism, were carried off with organizational aplomb and (for the UK hosts) unprecedented competitive success, reflecting their heavy investment in high performance sport but their concomitant failure to deliver on the promise of increased mass participation. They also delivered a magnificent new publicly-funded stadium to the wealthy owners of the Premier League’s West Ham United football club, and were instrumental in the construction of Westfield Stratford City—then Europe’s largest shopping mall—through which visitors to the main entrance of the Queen Elizabeth Olympic Park were obliged to transit.^42^ Moreover, the predictable and joyful sense of “liminality” associated with this and other SMEs—the “sense of celebration and a feeling that the social conditions and social barriers of everyday life are transcended during the time of the event”^43^—proved predictably transient and unpersuasive to an increasingly skeptical public still dealing with the fallout from the global financial crisis of 2008.

The Olympic end of this long cycle of hegemonic neoliberalism, punctuating and reinforcing the broader global trend, was delivered by the Rio Games in 2016. When the thirty-first Olympiad was awarded to Rio in 2009, defeating established “global cities”^44^ Chicago, Madrid, and Tokyo in the process, the decision sparked euphoria in a rapidly rising Brazil and seemed to embody the promise of upward mobility and a more inclusive global order, achieved through gentle reform rather than fundamental change. In this way, they seemed to validate the hegemonic promise of this order. By the time the Rio Games limped to their conclusion, however, Brazil was in the midst of a deep and protracted economic and political crisis and the Games had been drained of their rhetorical promise of wide-ranging developmental and environmental benefits to Rio’s deeply unequal populace. This outcome sharply discredited not only the earlier optimism surrounding the Rio Games but the model of inclusive neoliberalism on which the rise of the “BRICS”^45^ and other emerging economies was based. In the wake of these Games and followed by the Tokyo Games which, delayed by the COVID-19 pandemic, unfolded in bizarrely empty venues, the Olympics were once again in an existential crisis, and its pool of prospective hosts was once again confined to a small set of the world’s largest and richest cities, along with some of its most aggressively counter-hegemonic regimes.

## Beijing and beyond: Challenging hegemonic neoliberalism

Overlapping with the long cycle of the rise and decline of the hegemonic neoliberal Games was another trend, featuring a smaller set of revisionist, illiberal, and/or authoritarian regimes seeking to use the Olympics and other SMEs to cement their rising status and enhance the appeal of their alternative normative and political-economic models. The two most prominent examples are, of course, China and Russia.^46^

The Chinese government’s Olympic ambitions are well known and long standing. So, too, have been its efforts to advance a revised global order in which its alternative vision of the path to material capabilities, ideas of how social and political life should be organized, and conception of the global institutional fabric required to secure these revisionist objectives is at least accommodated. The impetus for China’s first Olympic bid, for the 2000 Games, has been directly attributed to Chinese leader Deng Xiaoping, and built directly on Beijing’s successful hosting of the 1990 Asian Games.^47^ China’s 1993 defeat by Sydney in its bid to host the 2000 Games was a serious blow to its collective pride, making the stakes that much higher when it bid again to host the Games in 2008.^48^ This time, the prospect of staging the Games in the world’s most populous and economically fastest-growing country proved irresistible to the IOC, alongside its corporate Olympic “Partners.” These material incentives were strongly reinforced by the IOC’s prime directive of achieving the universality of the Olympic movement. To those skeptics and critics who argued that awarding the Games to Beijing would in effect legitimate and enable its authoritarian form of government and its human rights abuses, advocates argued vaguely that the Games would incentivize further political openness, citing the Seoul example in support of this assumption.^49^ Indeed, Yihjye Hwang notes that “when Beijing won the bid for the 2008 Games, it promised the world that hosting the Olympics would improve its human rights record.”^50^

This hope or expectation proved, predictably, to be misplaced or perhaps more accurately, sharply contested. To be sure, there was no doubting the importance the Chinese Communist Party (CCP) regime invested in the Games, which were “seen and understood as part of an epochal shift in the nation’s relationship with the outside world, a process of change that had ignited the largest and fastest industrial revolution in history.”^51^ At the same time, the Games were underpinned by a conception of global order “at odds with the fuzzy cosmopolitanism of international human-rights regimes. China started from the principle that sovereignty and autonomy were the preconditions of entry into the international order, rather than subservience to international law”^52^—especially when that law was seen as vehicle for unjustified Western intrusions into its (and other non-Western regimes’) internal affairs.

In the run-up to the 2008 Games, these competing conceptions of global order and, in particular, the appropriate understanding of human rights and sovereignty within them were placed into sharp relief—most prominently by celebrity activism focused on China’s complicity in atrocity crimes in the Darfur region of Sudan, and by the mass protests over demands for self-determination in Tibet and a cluster of other human rights causes that dogged the Olympic torch relay as it made its way through London, Paris, San Francisco, and Delhi.^53^ The Chinese relay organizers seemed unprepared for these manifestations of large-scale opposition and the claims that underpinned them—reflecting the degree to which these organizers came from a different understanding of the appropriate norms of good international citizenship and world order. The torch relay protests generated widespread counter-protests from Chinese citizens in the diaspora, and a counter-discourse concerning what the Chinese government argued was the overwhelming human rights *achievements* of the PRC in lifting hundreds of millions of people out of poverty and advancing the right to development, without which (from its perspective) all other rights were moot.

At the Games themselves, launched with an awesome opening ceremony before an audience that included over one hundred heads of state and government,^54^ China modelled a very different version of organizational virtuosity and success from its capitalist counterparts. It mounted a spectacular event against a backdrop of iconic venues (including the striking National Stadium or “Bird’s Nest,” which was maintained as an expensive but underutilized tourist attraction following the closing of the Games) at a then-unprecedented official cost of more than $40 billion.^55^ Here, Lilach Gilady’s analysis of conspicuous consumption in anchoring claims to enhanced prestige and status is apt: the Chinese government was both able and entirely willing to mobilize and expend resources lavishly as an expression of its elevated standing, as well as a striking manifestation of the comparative strengths of its alternative model of political and economic organization. It was also able and willing to impose its will in order to do so—for example, by displacing an estimated 1.5 million people to make way for Olympic-linked urban development projects.^56^ The fact that so many heads of state were there for the Games’ opening ceremonies, moreover, provided a striking illustration of the Chinese government’s international convening power. In short, the discursive framing and monumental implementation of this event was an expression of a coherent alternative to the Western-centred model of hegemonic neoliberalism—an alternative that had underpinned China’s extraordinary developmental achievements.

A similar sequence and logic was apparent in the preparations for and hosting of the Sochi Winter Olympics in 2014. In this case, however, the objective of Vladimir Putin’s Olympic ambitions, orchestrated in conjunction with a supporting cast of wealthy oligarchs and state-linked corporations, was arguably less to advance a coherent alternative to the heretofore dominant model of hegemonic neoliberalism than to assert Russia’s autonomy from it—along with its reassertion of Great Power status within a reconstituted Eurasian sphere of influence. In the words of Kramareva and Grix, “Since Putin’s rise to power and a renewed…emphasis on Russia’s international stature, Russia has begun its reintegration into the shifting world order, first as a normal power and later, hurt by what it saw as Western arrogance and contempt, from the more assertive position (in which it) began playing host to a string of SMEs…”^57^ This analysis has of course been starkly reinforced by the Ukraine war launched by Russia in late February 2022.

As with the months preceding the Beijing Games, the most prominent flashpoint in the run-up to Sochi revolved around the contested terrain of human rights, with Russian legislators accused by Western human rights groups of having “roll(ed) back social and political advances (by adopting legislation that) helped to criminalize members of the LGBT community.”^58^ Rights advocates, primarily in Europe and North America, differed over whether to advocate a boycott in protest over these assaults on LGBT rights or to use the platform of the Games for active expressions of disapproval and resistance. They aligned, however, to mobilize a public and media outcry that sought to place Russia’s legislation beyond the pale. Russian officials, for their part, seemed unmoved by the protests and indeed revelled in their resistance to these efforts to impose “Western values,” reflecting the masculinist and heterosexist conception of Russian nationalism advanced and embodied by Vladimir Putin.

The distinctive features of the Winter Games are noteworthy in this context. Although bearing the cultural, political, and branding aura of Olympism, the Winter Games are much smaller in scale (with 2,780 athletes at Sochi versus 10,568 in London) and are dominated competitively by relatively wealthy northern and Western countries concentrated in Europe, North America, and East Asia.^59^ As a result, the protests decrying Russia’s homophobic legislation resonated particularly powerfully among the Winter Games’ Western core, but had little impact in the “majority world” of the Global South. In this sense, Russia (already increasingly at odds with the West politically) cared little about the protests and might have even gained credit among non-Western governments and peoples who resented the use of “rights talk” to discipline them, politically and economically.^60^

If the Winter Games had a considerably smaller reach in terms of the number and nationality of competitors as well as their global audience, however, this was not reflected in the eye-popping and prestige-seeking budget, nor the monumental infrastructural and venue developments undertaken for the Sochi Olympics. The Russian organizers blew through the previous high-water mark for Olympic spending reached in Beijing, with estimated expenditures of $55 billion that were massively publicly subsidized, and which left behind a spectacular but soon-after largely abandoned Sochi Olympic Park.^61^ Moreover, this already-tarnished legacy was further diminished by revelations of a massive state-sponsored doping scheme, implemented as part of a systematic effort to enable Russian athletes to reach the top of the Sochi medal table after their humbling eleventh-place standing at the 2010 Vancouver Winter Games. The revelations resulted in IOC’s half-hearted “suspension” of Russian athletes from international competition under the country’s flag and name^62^ for a four-year period (later, remarkably, halved to two years).^63^ Yet despite these excesses, the Games did not diminish the domestic popularity of President Putin, while the IOC’s felt need to retain the engagement of one of its largest and most powerful members meant that it was unwilling to take an appropriately hard line, further diminishing its credibility. The weak and enabling approach adopted by the IOC and other ISOs was further underscored when the Russian military forcibly and illegally annexed Crimea from Ukraine within days of the closing of the Sochi Winter Games and immediately prior to the start of the Sochi Paralympic Games, described by the International Paralympic Committee as “a stunning success, exceeding all expectations.^64^ Taken together, Sochi illustrated the extent to which illiberal populism had been consolidated in Russia and had become increasingly tolerated internationally.^65^ If not offering a coherent alternative to the Western-centred hegemonic order, Sochi embodied a highly visible and unapologetic challenge to it.

## Conclusion: Beijing 2022, the war in Ukraine, and world order interregnum^66^

This brings us to the latest chapter in Olympic hosting, with the events, statements, and actions surrounding the 2022 Winter Olympics in Beijing, followed almost immediately by the Russian invasion of Ukraine. Given their considerably smaller scale and audience, these Beijing Winter Games (with a comparatively modest projected budget of $3.9 billion but an actual cost estimated at up to $38 billion)^67^ were always anticipated to matter less to China than the 2008 Summer Games. They also reflected the declining image of the Olympic brand among Western countries, with a cluster of prospective European hosts withdrawing their bids and leaving only Beijing and Almaty, Kazakhstan to compete for hosting rights.^68^ Nevertheless, they were still “of great symbolic importance”^69^ to Communist Party general secretary Xi Jinping and the Chinese government. For China, their importance was amplified by the fact that they were the first Winter Games to be hosted in the same city as a previous Summer Games—China’s venerated capital. Moreover, they came at a pivotal time for the CCP, only months before its five-year Party Congress at which President Xi would seek to become the first leader since Mao Zedong to lead China for more than two five-year terms. Given these stakes, China warned that it would respond harshly to international efforts to tarnish or diminish the Games by other governments, media, or human rights organizations, among others; and under Xi’s “harsh authoritarian” regime^70^ China had already demonstrated its willingness to wield the country’s vast and growing political, military, and economic power punitively. Its expanding economic strength and sophistication, achieved through a very different political-economic model from its Western counterparts, alongside its rapid arms buildup has led many Western commentators to view China as a preeminent and growing strategic threat. For its part, China was eager to seize the opportunity of the Games to highlight its sophisticated technological and organizational capacity, including its ability to mount a mega-event amidst the lingering COVID-19 pandemic. In short, the Games were a prominent part of its ongoing effort to consolidate China’s international prestige.

At the same time, China’s human rights record, along with its record of commercial espionage among other internationally anti-social activities,^71^ had significantly worsened since it was chosen to host the Games. Most attention was focused on what a number of Western countries have declared to be a genocide against Muslim Uyghurs in Xinjiang province^72^ and the increasingly harsh enforcement of Hong Kong’s new security laws which have sharply diminished the freedoms previously enjoyed in the territory. This led 180 human rights groups to call for a boycott of the Games in February 2021, and two hundred to call on Olympic broadcasters to cancel their Olympic coverage in September 2021.^73^

Thus, China was once again drawing on the Olympics in its effort to reframe both the hierarchy and the balance of ideas, institutions, and material capabilities underpinning the increasingly frayed world order—while expressing much greater impatience and resentment towards foreign criticisms.^74^ Western states, on the other hand, were under heightened pressure from human rights and other advocates inside and outside their governments to take meaningful action to challenge the legitimacy and popular impact of these Games and, by extension, their Chinese hosts. The results of this sustained lobbying campaign were predictably limited, but included “diplomatic boycotts” (i.e., boycotts by official government representatives of the opening and closing ceremonies and other events) from the countries of the “Anglosphere” (the US, UK, Canada, Australia, and New Zealand^75^), along with another eight governments who stayed away for a variety of stated reasons. Television ratings were dramatically down from the previous Winter Games in Pyeongchang, South Korea,^76^ and corporate sponsors and broadcasters avoided the habitual, celebratory references to the host society, reducing any soft power benefits.^77^ At the same time, these effects were at least partially explained away by a new global wave of the COVID-19 infection linked to the Omicron variant, and China was able to claim an organizational success under exceptionally difficult circumstances. In short, while differences hardened and international prestige effects were diminished, the impact of international criticism was muted.

On the other hand, in the end the Games were dramatically overshadowed by the buildup to the Russian invasion of Ukraine. Launched only four days after the closing ceremonies and just before the start of the Paralympic Games, the invasion was quickly framed as a blatant breach of the Olympic Truce agreed to by the General Assembly of the United Nations.^78^ This in turn led the world’s leading ISOs—the IOC and FIFA among others—to a dramatic and belated reversal of their previous prevarications, and to a ban on Russian participation in international competition, including FIFA World Cup qualifying.^79^

While sport was therefore thrust, once again, into the international political spotlight, the instinctive reaction of many will be to conclude that the ramifications of this war highlight sport’s fundamentally trivial impact in the long process of world-order transition. Once again, it is the “high politics” of large-scale armed conflict that will be seen by many, understandably enough, as the fundamental driver of world order change—whatever direction this change ultimately takes. To do so, however, is to overlook the broader underpinnings of these processes, including the ramifications of SMEs and the organizations that govern them. It is to overlook, for example, the prominent role of sport in the resurgence of an aggressive and revanchist form of Russian nationalism under the leadership of Vladimir Putin, and the degree to which the hosting of key SMEs was used to project and reinforce this resurgence, domestically and internationally.^80^ It is also to overlook the degree to which the IOC, FIFA, and other ISOs, by lionizing Putin and failing to effectively punish anti-social behaviours such as systematic state-sponsored doping, normalized and enabled the continued expansion of his regime’s ambitions. While it is therefore clearly a mistake to overstate the role of SMEs and ISOs in these processes, it is equally in error to understate and overlook them.

Moreover, this prominent and peculiar strain of transnational non-state governance and the practices they oversee will continue to be a key window on efforts to both advance and inhibit processes of hegemonic contestation and transition. For example, faced with the declining appeal of SME hosting, the IOC has now locked into a series of Olympiads in major Western countries and global cities, including Paris (2024), Los Angeles (2028), and Brisbane (2032). Yet despite this apparent Western “tilt” (or indeed because of it), the Organization will be at pains to continue to find ways to express its “universality,” while struggling with the difficult and contested normative terrain of human rights.^81^ Its efforts to do so will be a key marker of the bases upon which a new post-hegemonic order will either emerge or flounder.

In short, the Olympics specifically, and SMEs generally, are a significant and understudied element of world-order making, sustaining, and remaking. International studies scholars owe them closer attention.

## Notes

1. See Ronald J. Deibert, “Subversion Inc.: The age of private espionage,” *Journal of Democracy* 33, no. 2 (2022): 28–44.

2. See Robert Cox, “Social forces, states, and world orders: Beyond international relations theory,” in Robert Cox with Timothy Sinclair, eds.*, Approaches to World Order* (Cambridge: Cambridge University Press), 85–123. It is important to note that even relatively stable hegemonic orders are never free from the shadow of coercion and domination, particularly towards the margins of the system within and between countries.

3. Cox, “Social forces, states, and world orders,” 87–91.

4. Milan Babic, “Let's talk about the interregnum: Gramsci and the crisis of the liberal world order,” *International Affairs* 96, no. 3 (2020): 767–786.

5. Susan Brownell, “Human rights and the Beijing Olympics: Imagined global community and the transnational public sphere,” *The British Journal of Sociology* 63, no. 2 (2012): 306–327.

6. The “multiple personalities” of the contemporary IOC mean that it is at once akin to an international diplomatic organization, a civil society-based social movement, and a multinational corporation. See Byron Peacock, “A secret instinct of social preservation’: Legitimacy and the dynamic (re)constitution of Olympic conceptions of the ‘good,’” *Third World Quarterly* 32, no. 3 (2011): 477–502.

7. The Olympics are one of two “first order” SMEs, along with the FIFA World Cup (to which reference will be made later in the paper). Events with a less extensive but nevertheless global reach—for example, the Rugby World Cup or the Commonwealth Games—are typically identified as second order events, and regional games (e.g., the Pan American, Asian, or All Africa Games) are seen as third order events.

8. Defined by Lilach Gilady, following Thorstein Veblen, as “conspicuous acts of consumption as public displays of one’s social standing.” See Gilady, *The Price of Prestige: Explaining Conspicuous Consumption in International Relations* (Chicago: University of Chicago Press, 2018), 14.

9. See for example David Black, “‘Not cricket': The effects and effectiveness of the sport boycott,” in Neta Crawford and Audie Klotz, eds., *How Sanctions Work: Lessons from South Africa* (London: Macmillan), 1999.

10. For a small sample of this voluminous literature, see Jules Boykoff, *Activism and the Olympics: Dissent at the Games in Vancouver and London* (New Brunswick, NJ: Rutgers University Press, 2014); John Gold and Margaret M. Gold, *Olympic Cities: City Planning, Agendas, and the World’s Games, 1896-2016*, 2^nd^ ed. (London: Routledge, 2011); David Goldblatt, *The Games: A Global History of the Olympics* (London: Norton, 2016); Jonathan Grix, ed., *Leveraging Legacies from Sports Mega-Events: Concepts and Cases* (London: Palgrave Macmillan, 2014); Allen Guttman, *The Olympics: A History of the Modern Games* (Chicago: University of Illinois Press, 1992); John Horne and Gary Whannel, *Understanding the Olympics*, 2^nd^ ed. (London: Routledge, 2016).

11. Bruce Kidd, “Canada needs a two-track strategy for hosting international games,”*Policy Options/Options Politiques*, 1 December 2009, https://policyoptions.irpp.org/fr/magazines/the-2010-olympics/canada-needs-a-two-track-strategy-for-hosting-international-games/ (accessed 28 January 2023).

12. For example, Goldblatt, *The Games*, and John Hoberman, “Toward a theory of Olympic internationalism,” *Journal of Sport History* 22, no. 1 (1995): 1–37.

13. For a partial exception, see Richard Gruneau and John Horne, eds., *Mega-Events and Globalization: Capital and Spectacle in a Changing World Order* (London: Routledge, 2015); and Peacock, “’A secret instinct of social preservation.’”

14. For an influential application of this distinction to the field of development, see Michael Cowen and Robert Shenton, *Doctrines of Development* (London: Routledge, 1996).

15. Beginning, infamously, with the 1936 Berlin Olympics—a propaganda coup for Adolf Hitler’s Nazi regime.

16. See Richard Arnold, “Sport and official nationalism in modern Russia,” *Problems of Post-Communism* 65, no. 2 (2018): 129–141; James Riordan, “Moscow 1980,” in John E. Findling and Kimberly Pelle, eds., *Historical Dictionary of the Modern Olympic Movement* (Westport, CT: Greenwood, 1996); Goldblatt, *The Games*, 303–309; and Nina Kramareva and Jonathan Grix, “’War and peace’ at the 1980 Moscow and 2014 Sochi Olympics: The role of hard and soft power in Russian identity,” *The International Journal of the History of Sport* 35, no. 14 (2018): 1407–1427.

17. James Riordan, “The role of sport in Soviet foreign policy.” *International Journal* 43, no. 4 (1988): 569–595.

18. Christopher Hill, *Olympic Politics, 2nd ed*. (Manchester: Manchester University Press, 1996), 122–123.

19. Kramareva and Grix, “’War and Peace,’” 1413.

20. Martin Barry Vinokur, *More than a Game: Sport and Politics* (Westport, CT: Greenwood, 1988), 116–120.

21. One consequence being that some vociferous governmental supporters of the boycott, including Britain’s Margaret Thatcher, could not prevent the British Olympic Committee from sending a British team to compete—though they could prevent it from marching under the Union Jack. On the resilient myth of sport’s autonomy from politics, see Heather Dichter, “The diplomatic turn: The new relationship between sport and politics,” *International Journal of the History of Sport* 38, nos. 2–3 (2021): 247.

22. Riordan, “Moscow 1980,” 164.

23. See, for example, Kim Nossal, “International sanctions as international punishment,” *International Organization* 43, no. 2 (1989): 301–322; and Black, “‘Not cricket.’”

24. Riordan, “Moscow 1980,” 166.

25. Hill, *Olympic Politics*, *2*^*nd*^*ed.,* 138–160; and Wayne Wilson, “Los Angeles 1984,” in John E. Findling and Kimberly Pelle, eds., *Historical Dictionary of the Modern Olympic Movement* (Westport, CT: Greenwood, 1996).

26. It is arguably too simplistic to portray the Soviet-led boycott in “tit-for-tat” terms, as has often been done. Nevertheless, the boycott was competitively significant, with many of the world’s then-leading sporting powers eschewing the Games, and it placed into sharp relief the comparative resilience of the two rivals in responding to these major disruptions. See Philip D’Agati, *The Cold War and the 1984 Olympic Games: A Soviet-American Surrogate War* (New York: Palgrave, 2013).

27. Goldblatt, *The Games*, 210–212.

28. On Samaranch and his legacy, see Jeré Longman, “Juan Antonio Samaranch, who transformed the Olympics, dies at 89.” *New York Times*, 10 April 2010, https://www.nytimes.com/2010/04/22/sports/22samaranch.html (accessed 14 January 2023).

29. Though to date, only two African bids have been made, from the two extremes of the continent—Cape Town, South Africa and Cairo, Egypt. I am primarily focusing in this paper on the virtually universal Summer Games; the much smaller and geographically-constrained Winter Games tell a related but distinct story that comes into play in the third phase discussed below.

30. See Grix, *Leveraging Legacies*.

31. Boykoff, *Activism and the Olympics*.

32. Nagoya, Japan was the only other short-listed bidder, and its prospects were scuppered by vigorous local protests. See David R. Black and Shona Bezanson, “The Olympic Games, human rights, and democratisation: Lessons from Seoul and implications for Beijing,” *Third World Quarterly* 25, no. 7 (2004): 1245–1261; and Goldblatt, *The Games,* 319–325.

33. See David Black and Byron Peacock, “Catching up: Understanding the pursuit of major games by rising developmental states,” *International Journal of the History of Sport* 28, no. 16 (2011): 2270–2287.

34. Goldblatt, *The Games*, 321.

35. Goldblatt, *The Games,* 344–345; also Horne and Whannel, *Understanding the Olympics*, 204–205.

36. See, for example, Gordon Waitt, “The ‘green’ Games Sydney 2000 played,” in John Nauright and Kimberley Schimmel, eds., *The Political Economy of Sport* (London: Palgrave, 2005), 183–207.

37. David Rowe, “The Sydney Olympics: How did ‘the best Games ever’ change Australia?” *The Conversation*, 13 September 2020, https://theconversation.com/the-sydney-olympics-how-did-the-best-games-ever-change-australia-145926 (accessed 14 January 2023).

38. Rowe, “The Sydney Olympics.”

39. Most closely associated with the UK’s “New Labour” governments led by Tony Blair and Gordon Brown, but also the Clinton administration in the United States and, in the view of some, the Chrétien Liberal government in Canada.

40. Wolfram Manzenreiter, “Magical thought and the legacy discourse of the 2008 Beijing Games,” in Jonathan Grix, ed., *Leveraging Legacies from Sports Mega-Events: Concepts and Cases* (London: Palgrave Macmillan, 2014).

41. Goldblatt, *The Games*, 384–385.

42. Horne and Whannel, *Understanding the Olympics*, 227–231.

43. Laurence Chalip, “From legacy to leverage,” in Jonathan Grix, ed., *Leveraging Legacies from Sports Mega-Events: Concepts and Cases* (London: Palgrave Macmillan, 2014), 5.

44. On the rising salience of global cities, see Saskia Sassen, *The Global City: New York, London, Tokyo* (Princeton: Princeton UP, 2002).

45. Brazil, Russia, India, China, and South Africa.

46. Though it is noteworthy that other more-and-less illiberal regimes, including Turkey, Hungary, Kazakhstan, Cuba, and Azerbaijan, have mounted Olympic bids in the new millennium, while fewer and fewer Western cities and countries have been prepared to bid. See *The Economist,* “International sporting events are increasingly held in autocracies,” 17 November 2022, https://www.economist.com/graphic-detail/2022/11/17/international-sporting-events-are-increasingly-held-in-autocracies (accessed 14 January 2023).

47. See Shiming Luo and Fuhua Huang, “China’s Olympic dream and the legacies of the Beijing Olympics,” *The International Journal of the History of Sport* 30, no. 4 (2013): 447–448.

48. As Lilach Gilady discusses in *The Price of Prestige*, collective pride and prestige are closely interconnected in international relations.

49. See Black and Bezanson, “The Olympic Games.”

50. Yihjye Hwang, “Olympiad, a place of linguistic struggle—the discursive constitution of ‘human rights’ in the 2008 Beijing Olympics,” *Sport in Society* 13, no. 5 (2010): 870. Susan Brownell, however, argues that this “promise” was, in fact, highly qualified and underpinned by an alternative Chinese vision of the Games as a vehicle to advance its normative conception of *humanism*, as distinct from legalistic Western understandings of *human rights*. See Brownell, “Human rights and the Beijing Olympics: imagined global community and the transnational public sphere,” *The British Journal of Sociology* 63, no. 2 (2012): 313.

51. See Goldblatt, *The Games,* 400; and Hwang, “Olympiad,” 863 on the “hugely popular” view of the Olympics among Chinese citizens.

52. Goldblatt, *The Games,* 401.

53. For example, see Horne and Whannel, *Understanding the Olympics,* 205–206.

54. Hwang, “Olympiad,” 867.

55. By comparison, the London Olympic Games four years later were estimated to have cost just over $13 billion US dollars—although the costing of modern Olympic Games is notoriously slippery. See James McBride and Melissa Manno, “The economics of hosting the Olympic Games,” *Council on Foreign Relations*, 14 December 2021, https://www.cfr.org/backgrounder/economics-hosting-olympic-games#:∼:text=Beijing's_2008_Summer_Olympics_generated,with_2418_billion_in_costs (accessed 14 January 2023).

56. Manzenreiter, “Magical thought and the legacy discourse,” 124.

57. Kramareva and Grix, “’War and Peace,’” 1415.

58. Judy Davidson and Mary G. McDonald, “Rethinking human rights: the 2014 Sochi Winter Olympics, LGBT protections and the limits of cosmopolitanism,” *Leisure Studies* 37, no. 1 (2018): 64.

59. Jung Woo Lee argues that in contrast to the Summer Games, the Winter Games seem to “reinforce Western cultural hegemony and white privilege.” See Lee, “Olympic Winter Games in non-Western cities: State, sport and cultural diplomacy in Sochi 2014, PyeongChang 2018, and Beijing 2022,” *The International Journal of the History of Sport* 38, nos. 13–14 (2021): 1494.

60. Donaldson and McDonald, “Rethinking human rights.”

61. Martin Müller, “After Sochi 2014: Costs and impacts of Russia’s Olympic Games,” *Eurasian Geography and Economics* 55, no. 6 (2014): 628–655; and Jason Ponic, “Abandoned Olympic venues,” How They Play*,* 23 March 2020, https://howtheyplay.com/olympics/AbandonedOlympicVenues (accessed 14 January 2023).

62. They defiantly competed, unimpeded, under the ROC (Russian Olympic Committee) moniker.

63. Sean Ingle, “Decision to halve Russia's Wada doping ban met with disbelief and anger,” *The Guardian*, 17 December 2020, https://www.theguardian.com/sport/2020/dec/17/russia-doping-ban-halved-but-name-and-flag-barred-from-next-two-olympics-court-of-arbitration-for-sport-world-anti-doping-agency (accessed 14 January 2023). On the high political priority placed on Russia’s success in the 2014 medal table, see for example Michal Marcin Kobierecki, “Russia and its international image: From Sochi Olympic Games to annexing Crimea,” *International Studies: Interdisciplinary Political and Cultural Journal* 18, no. 2 (2016): 174–175. The extraordinary doping scheme undertaken to support it was dramatically recounted in the 2017 documentary *Icarus*. See Bryan Fogel, dir., *Icarus* (Los Angeles CA: Diamond Docs, 2017), https://www.netflix.com/title/80168079 (accessed 28 January 2023).

64. International Olympic Committee, “Sochi 2014 Paralympic Winter Games,” Sochi 2014 Paralympic Winter Games, 7-16 March 2014, https://www.paralympic.org/sochi-2014 (accessed 14 January 2023). See also Kobierecki, “Russia and its international image,” and Kramareva and Grix, “’War and Peace.’”

65. This toleration was strongly reinforced by the highly positive reception of the Russian-hosted 2018 FIFA World Cup, highlighting “sport’s capacity for image rehabilitation”; see Cathal Kelly, “Russia’s World Cup and sport’s capacity for image rehabilitation,” *The Globe and Mail*, 13 July 2018, https://www.theglobeandmail.com/sports/article-russias-world-cup-and-sports-capacity-for-image-rehabilitation/ (accessed 14 January 2023).

66. For a Gramscian interpretation of world order “interregnum,” during which (in Gramsci’s words) “the old is dying and the new cannot be born,” see Babic, “Let’s talk about the Interregnum.”

67. See “How Beijing spent billions more than the official Olympic budget/true cost,” Business Insider, 3 February 2022, https://www.youtube.com/watch?v=cvr2Keh64cQ (accessed 23 March 2022).

68. Jean-Loup Chappelet, “Winter Olympic referendums: Reasons for opposition to the Games,” *International Journal of the History of Sport* 38, nos. 13–14 (2021): 1369–1384; and John McAloon, “Agenda 2020 and the Olympic movement,” *Sport in Society* 19, no. 6 (2016): 767–785.

69. *The Economist,* “China is determined to make the Winter Olympics go smoothly,” 26 January 2022, https://www.economist.com/china/china-is-determined-to-make-the-winter-olympics-go-smoothly/21807403 (accessed 28 January 2023).

70. *The Economist,* “Faster, higher, bossier.” See also *The Economist,* “The war in Ukraine is going to change geopolitics profoundly,” 5 March 2022, https://www.economist.com/briefing/2022/03/05/the-war-in-ukraine-is-going-to-change-geopolitics-profoundly (accessed 28 January 2023).

71. Such as the incarceration of Canada’s “two Michaels” in thinly-veiled retaliation for Canada’s detention of Huawei’s chief financial officer Meng Wanzhou on the basis of a US extradition request—see CBC News, “The Meng Wanzhou Huawei saga: A timeline,” 24 September 2021, https://www.cbc.ca/news/meng-wanzhou-huawei-kovrig-spavor-1.6188472 (accessed 28 January 2023).

72. See Darren Byler, “China’s internment camps in Xinjiang are a horror. Survivors and participants alike must reconcile with the truth,” *Globe and Mail*, 23 October 2021, https://www.theglobeandmail.com/opinion/article-chinas-internment-camps-in-xinjiang-are-a-horror-survivors-and/ (accessed 23 October 2021).

73. Macintosh Ross and Helen Jefferson Lenskyj, “A broadcast boycott is the last chance to mount serious resistance against the Beijing Olympic Games,” *The Conversation*, 19 September 2021, https://theconversation.com/a-broadcast-boycott-is-the-last-chance-to-mount-serious-resistance-against-the-beijing-olympic-games-167757 (accessed 14 January 2023).

74. For example, see Megan Walsh, “The Beijing Olympics feels like a curtain raiser for China’s defiant global hegemony,” *The Globe and Mail*, 28 January 2022, https://www.theglobeandmail.com/opinion/article-the-winter-olympics-as-metaphor-for-how-the-ccp-writes-its-narratives/ (accessed 28 January 2023).

75. Which along with several others said no government officials were attending because of concerns over COVID-19.

76. Simon Houpt, “CBC ratings for Beijing Olympic Games falling short amid Canadian ambivalence,” *The Globe and Mail*, 12 February 2022, https://www.theglobeandmail.com/sports/olympics/article-cbc-ratings-for-beijing-olympic-games-falling-short-amid-canadian/ (accessed 28 January 2023).

77. On SMEs and the quest for “soft power,” see, for example, Jonathan Grix and Paul Brannagan, “Of mechanisms and myths: Conceptualizing states’ ‘soft power’ strategies through sports mega-events,” *Diplomacy and Statecraft* 27, no. 2 (2016): 251–272.

78. On the now habitual “Olympic Truce”, see International Olympic Committee, “UN General Assembly adopts Olympic Truce for Beijing 2022, highlighting the contribution of sport to the promotion of peace and solidarity,” 2 December 2021, https://olympics.com/ioc/news/un-general-assembly-adopts-olympic-truce-for-beijing-2022 (accessed 14 January 2023). On the impact of the imminent invasion on the Beijing Games, see James Griffiths, “Vladimir Putin endorsed Xi Jinping’s Olympics, and then completely overshadowed them,” *The Globe and Mail*, 18 February 2022, https://www.theglobeandmail.com/sports/olympics/article-vladimir-putin-endorsed-xi-jinpings-olympics-and-then-completely/ (accessed 28 January 2023).

79. Paul MacInness and agencies, “Russia suspended from all FIFA and UEFA competitions until further notice.” *The Guardian*, 28 February 2022, https://www.theguardian.com/football/2022/feb/28/fifa-and-uefa-suspend-russian-clubs-and-teams-from-world-cup-and-all-competitions (accessed 14 January 2023).

80. Arnold, “Sport and official nationalism in modern Russia.”

81. See Jean-Loup Chappelet, “The Olympics’ evolving relationship with human rights: An ongoing affair,” *Sport in Society* 25, no. 1 (2022), 1–22.

